# Common Cancer Types and Risk of Stroke and Bleeding in Patients With Nonvalvular Atrial Fibrillation: A Population‐Based Study in England

**DOI:** 10.1161/JAHA.123.029423

**Published:** 2023-09-26

**Authors:** Alyaa M. Ajabnoor, Rosa Parisi, Salwa S. Zghebi, Darren M. Ashcroft, Corinne Faivre‐Finn, Charlotte Morris, Mamas A. Mamas, Evangelos Kontopantelis

**Affiliations:** ^1^ Department of Pharmacy Practice, Faculty of Pharmacy King Abdulaziz University Jeddah Saudi Arabia; ^2^ Division of Informatics, Imaging and Data Sciences, School of Health Sciences, Faculty of Biology, Medicine and Health, Manchester Academic Health Science Centre (MAHSC) University of Manchester Manchester United Kingdom; ^3^ Division of Population Health, Health Services Research and Primary Care, School of Health Sciences, Faculty of Biology, Medicine and Health, Manchester Academic Health Science Centre (MAHSC) University of Manchester Manchester United Kingdom; ^4^ Centre for Pharmacoepidemiology and Drug Safety, Division of Pharmacy and Optometry, School of Health Sciences, Faculty of Biology, Medicine and Health University of Manchester Manchester United Kingdom; ^5^ National Institute for Health and Care Research (NIHR) Greater Manchester Patient Safety Research Collaboration (PSRC) University of Manchester Manchester United Kingdom; ^6^ The Christie NHS Foundation Trust and The University of Manchester Manchester United Kingdom; ^7^ Keele Cardiovascular Research Group, Centre for Prognosis Research, Institute for Primary Care and Health Sciences Keele University Keele United Kingdom

**Keywords:** atrial fibrillation, bleeding, cancer, oral anticoagulant, stroke, Epidemiology, Atrial Fibrillation, Anticoagulants, Ischemic Stroke, Intracranial Hemorrhage

## Abstract

**Background:**

The association between cancer and stroke or bleeding outcomes in atrial fibrillation is unclear. We sought to examine how certain types of cancer influence the balance between stroke and bleeding risk in patients with nonvalvular atrial fibrillation (NVAF).

**Methods and Results:**

We estimated stroke and bleeding risk among adult patients with NVAF and certain types of cancer (breast, prostate, colorectal, lung, and hematological cancer) from 2009 to 2019 based on data from the UK Clinical Practice Research Datalink GOLD and Aurum databases. The control group included patients with NVAF only. Of 177 065 patients with NVAF, 11379 (6.4%) had cancer (1691 breast, 3955 prostate, 1666 colorectal, 2491 hematological, and 1576 lung). Compared with patients without cancer, stroke risk was higher in patients with breast cancer (adjusted hazard ratio [aHR], 1.20 [95% CI, 1.07–1.35) and with prostate cancer (aHR, 1.11 [95% CI, 1.01–1.12) if diagnosed within 6 months before NVAF. The risk of bleeding was increased in subjects with hematological cancer (aHR, 1.55 [95% CI, 1.40–1.71]), lung cancer (aHR, 1.49 [95% CI, 1.25, 1.77]), prostate cancer (aHR, 1.38 [95% CI, 1.28–1.49]), and colorectal cancer (aHR, 1.36 [95% CI, 1.21–1.53]), but not for subjects with breast cancer. The more recent the cancer diagnosis before NVAF diagnosis (within 6 months), the higher the risk of bleeding.

**Conclusions:**

Breast and prostate cancer are associated with increased stroke risk, whereas in some cancer types, the risk of bleeding seemed to exceed stroke risk. In these patients, prescribing of oral anticoagulant should be carefully evaluated to balance bleeding and stroke risk.

**Nonstandard Abbreviations and Acronyms**
CPRD: Clinical Practice Research Datalink
HES: Hospital Episodes Statistics
OAC: oral anticoagulant
ONS: Office for National Statistics

**Clinical Perspective**

**What Is New?**

- In patients with nonvalvular atrial fibrillation, risk of stroke is increased in patients with breast cancer and prostate cancer compared with patients without cancer, after adjusting for comorbidities and exposure to oral anticoagulant or aspirin.
- Bleeding risk is increased in patients with history of hematological cancer, followed by lung cancer, prostate cancer, and colorectal cancer.
- In patients with nonvalvular atrial fibrillation and cancer, prescribing of oral anticoagulant should be carefully evaluated to balance bleeding and stroke risk.

**What Are the Clinical Implications?**

- In certain types of cancer, such as hematological and lung malignancies, the risk of bleeding could exceed that of stroke in case of nonvalvular atrial fibrillation.
- These clinical data motivate future clinical and translational studies to determine the true clinical benefit of anticoagulation in cancer associated atrial fibrillation, and develop accurate risk assessment tools that consider specific types of cancer.

Atrial fibrillation (AF) is the most common sustained cardiac arrhythmia and an independent risk factor for stroke, increasing its risk by 5‐fold.^1^ In the general population, the risk of stroke in patients with AF is mainly assessed by means of the CHA_2_DS_2_‐VASc score, which consists of 8 categories, with points given for each of the following: congestive heart failure, hypertension, age ≥75 years (×2 points), diabetes mellitus, prior stroke or transient ischemic attack (TIA) or thromboembolism (×2 points), vascular disease, age 65 to 74 years, and sex category and the decision to prescribe oral anticoagulant therapy is based on the balance between future risk of stroke and the risk of major bleeding. AF is known to be associated with cancer, a relationship likely confounded by common cardiovascular risk factors, neoplastic infiltration of the atrial tissue, the effects of radiotherapy and systemic anticancer therapies as well as cancer‐induced inflammation.^2^, ^3^, ^4^, ^5^

In the cancer population, the risk of venous thromboembolic disease, and the risk of arterial thromboembolism including stroke, is increased in most cancer types, particularly in the period after diagnosis.^6^, ^7^ This is believed to be secondary to the hypercoagulable state associated with cancer and the pro‐thrombotic effect increased by some chemotherapies that could also increase the risk of bleeding.^8^ These factors are not considered by the validated thromboembolic risk assessment score CHA_2_DS_2_‐VASc, nor the HAS‐BLED score, which consists of 9 points, one for each of the following: hypertension, abnormal kidney or liver function (1 point each), stroke, history of bleeding or predisposition, labile international normalised ratio (INR), elderly (>65 years), and drugs/alcohol concomitantly (1 point each) bleeding risk score that drive decisions around the prescription of anticoagulants.^9^ Thus, the value of these assessment scores is unknown in the cancer population, and therefore, the decision to start anticoagulant therapy is usually individualized according to cancer type, prognosis of cancer, patient preference, drug–drug interactions, and physical performance.

Patients with AF and history of cancer are often excluded from clinical trials, which makes it challenging to apply evidence‐based management in this high‐risk group. The association between cancer and stroke or bleeding outcomes in patients with AF is still unclear. Furthermore, in post hoc analyses, the incidence of stroke and bleeding reported in the small numbers of patients with cancer enrolled in randomized controlled trials have reported varying results.^10^, ^11^, ^12^ Therefore, there are limited data around the distribution of stroke and bleeding risk in the cancer population, and how it differs from the general AF population. This will enable better understanding of the prognostic impact of cancer on patients with AF, especially when considering the onset of AF after cancer diagnosis and type of cancer. In this study, we aimed to use electronic health record data to compare stroke and bleeding risk in patients with nonvalvular atrial fibrillation (NVAF) with common cancer types, to those with no cancer.

## METHODS

### Study Design and Data Source

We conducted a population‐based retrospective cohort study using electronic health record data obtained from 2 databases: the Clinical Practice Research Datalink (CPRD) GOLD, collecting data from general practices in the UK using the Vision clinical computer system; and CPRD Aurum, collecting data from general practices in England only using the EMIS clinical computer system.^13^, ^14^ CPRD is broadly representative of the UK population, CPRD GOLD represents around 7% of the UK population, and CPRD Aurum represent around 13%, respectively.^13^, ^14^ The study only included data from English practices agreeing to data linkages to the 2015 Index of Multiple Deprivation,^15^ Hospital Episodes Statistics (HES) for admitted patients, and Office for National Statistics (ONS) death registration.^13^, ^14^ ONS data are considered the gold standard for mortality data in the UK and contain the date, place, and cause of death. While HES data sets include Admitted Patient Care data that contain details of all admissions to, or attendances at English health care providers of the National Health Service, including acute hospital trusts, primary care trusts, and mental health trusts.^13^, ^14^ The data was requested via application to the Clinical Practice Research Datalink and approved by the CPRD's Independent Scientific Advisory Committee (protocol number: 20_198R). The requirement for informed consent was waived because these databases are anonymized following strict confidentiality guidelines before being distributed for research purposes. The raw data underlining the results presented in the study are subject to CPRD's Research Data Governance process (https://cprd.com/data‐access, contact enquiries@cprd.com). Because of data license restrictions, study data will not be made available upon request.

### Study Population

**Figure 1:**
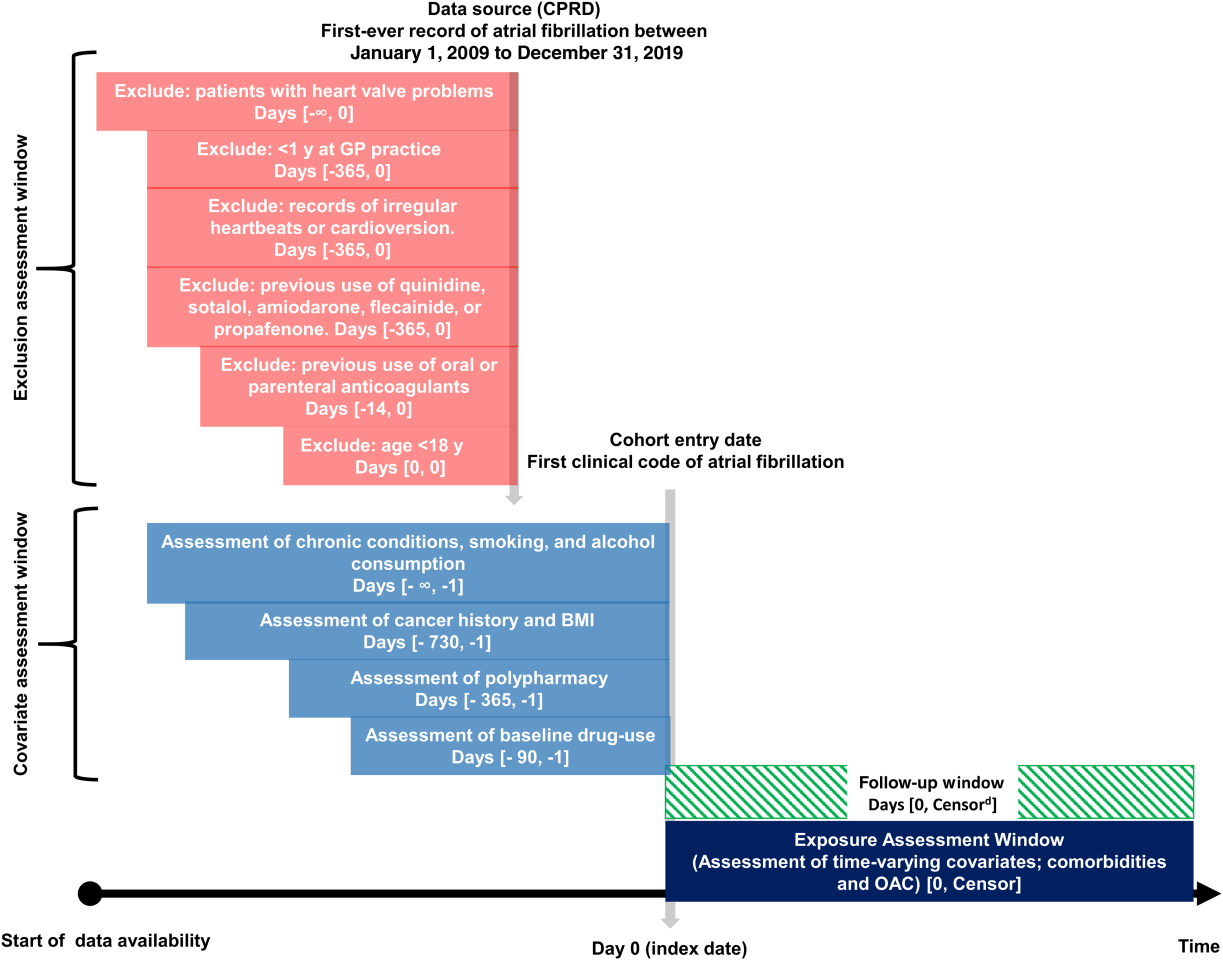
Visualization of study design and patient follow‐up. BMI indicates body mass index; CPRD, clinical practice datalink; GP, general practice; and OAC, oral anticoagulant.

The defined study cohorts consisted of patients with first‐ever record of NVAF between January 1, 2009 and December 31, 2019. Eligible patients were adults aged ≥18 years and registered in a general practice in England for at least 1 year before NVAF diagnosis. Patients with existing valvular pathology before AF diagnosis were excluded. Additional exclusion criteria were applied within a lookback period of 12 months before NVAF diagnosis: records of irregular heartbeats or cardioversion, records of atrial flutter alone with no mention of NVAF, and previous use of quinidine, sotalol, amiodarone, flecainide, or propafenone. Patients with history of cancer were defined as patients with a diagnostic code for cancer within 2 years before the index date of NVAF diagnosis. We focused on the most common cancer types diagnosed in England^16^: breast, prostate, colorectal, and lung cancer, which are also associated with cardiovascular disease.^17^ We have also included patients with hematological malignancy, because it is known to increase bleeding risk.^18^ Only patients with diagnosis of these types of cancer were included in the cancer group, and patients with other types of cancer were excluded from the analysis. The comparison group included patients with NVAF without a diagnosis of cancer before index date. Records for NVAF and cancer were identified using Read codes alone in CPRD GOLD or using both SNOMED/EMIS and Read codes in CPRD Aurum. Cancer diagnosis by type was supplemented with HES data, using *International Classification of Disease, Tenth Revision* (*ICD‐10*) codes. Diagnostic codes were independently reviewed by an expert clinician (M.A.M.), and medication lists were reviewed by the first author (A.M.A.). The clinical codes used to produce the data for this study can be found in https://clinicalcodes.rss.mhs.man.ac.uk.^19^ Patients' follow‐up started from the NVAF diagnosis (index date) and continued until the earliest of the end of study observation period (December 31, 2019), patients transferred out of practice, last collection date for the practice, occurrence of the outcome of interest (stroke event or major bleeding), cancer diagnosis (in case of the comparison population), or death (Figure 1).

### Exposure to Oral Anticoagulants

Patients prescribed an oral anticoagulant (OAC) after a diagnostic record for NVAF were identified as OAC initiators. Patients were excluded if they had received oral or parenteral anticoagulants >14 days before NVAF diagnosis. OACs available in the UK were identified from the British National Formulary. Prescribing data are recorded using the Gemscript product code system in GOLD and dictionary of medicines and devices prescribing codes in Aurum database.^13^, ^14^ Exposure to OAC included either warfarin or non‐vitamin K antagonist oral anticoagulant (NOAC) such as dabigatran, rivaroxaban, apixaban, and edoxaban. Continuous exposure to OAC was defined as sequential prescriptions of the same drug within a grace period of 30 days after the expected end of the previous prescription. A gap of 30 days or less between the end of days of supply of one prescription and the next was assumed to be a continuous treatment episode. In case of NOACs, the quantity issued was estimated by dividing the number of tablets prescribed by the approved number of daily doses (twice daily for dabigatran and apixaban, and once a day for rivaroxaban and edoxaban). However, if the quantity of the NOAC issued was not available, the quantity was estimated using the mean number of NOAC tablets prescribed for the same drug for that patient or the overall mean for that drug if patient‐specific data were not available. Since precise dosages for warfarin were not available because they vary according to international normalized ratio (INR) measurements and are not consistently recorded in general practice, the median time between all previous sequential prescriptions of warfarin for each patient was used to estimate days of supply. INR measurements, if reported, were treated as an indicator for warfarin exposure and therefore treated in the same way as prescriptions. Additionally, if a patient was prescribed aspirin with no OAC, an exposure status of aspirin‐only was assigned to those patients.

### Outcomes

The primary outcomes of the study were ischemic stroke/transient ischemic attack defined as the earliest record after index date in either CPRD, HES, and ONS records. In addition, major bleeding events defined as a hospital record in HES data after the index date of NVAF diagnosis. Major bleeding could be (1) bleeding occurring at a critical site (ie, intracranial, intraspinal, intraocular, pericardial, intra‐articular, intramuscular, and retroperitoneal), (2) bleeding that led to hospitalization, or (3) fatal bleeding.^20^ All outcomes were identified using CPRD codes for primary care events, *ICD‐10* codes for HES, or ONS records.

### Covariates

We extracted baseline information on the following demographic and clinical risk factors: age, sex, body mass index (BMI), smoking and alcohol consumption status, ethnicity, Index of Multiple Deprivation in quintile, time from cancer diagnosis to NVAF diagnosis, comorbidities, and baseline medications. Comorbidities were also defined according to the Charlson comorbidity index.^21^ Ethnicity was extracted from HES records and categorized into 4 main ethnic groups: White, Black (including Black African, Black Caribbean, and other Black), Asian (including Chinese, Indian, Pakistani, Bangladeshi, and other Asian), and other ethnicities (including mixed ethnicities). Polypharmacy was assessed at baseline during 1 year before nonvalvular NVAF diagnosis using the common definition of the concomitant use of ≥5 medications.^22^ Risks for stroke and bleeding were assessed according to CHA_2_DS_2_‐VASc and HAS‐BLED scores, respectively. Since INR measurements are not consistently reported in CPRD, a modified HAS‐BLED score was used, which does not include the INR element and consists of 8 points. According to the European Society of Cardiology guidelines.^23^ OAC prescribing is considered with a CHA_2_DS_2_‐VASc score of 1 in men, or 2 in women, and is recommended with a score of ≥2 in males, or ≥3 in females. Therefore, patients were classified according to their stroke risk into low risk if CHA_2_DS_2_‐VASc score is equal to 0 in men, or 1 in women; intermediate risk if CHA_2_DS_2_‐VASc score equal 1 in men, or 2 in women, and high risk if CHA_2_DS_2_‐VASc score ≥2 in men, or ≥3 in women. Chronic conditions such as diabetes, hypertension, ischemic heart disease, myocardial infarction, heart failure, liver disease, chronic kidney disease, anemia and dementia were examined at baseline and as time‐varying covariates throughout follow‐up. Drugs used at baseline were explored if a patient received at least 1 prescription of a certain drug within 90 days before AF diagnosis. These included drugs for common chronic condition and systemic anticancer therapies if reported in primary care records.

### Statistical Analysis

All data extracted from CPRD GOLD and CPRD Aurum databases were combined before analysis. Descriptive statistics were used to analyze the baseline demographic and clinical characteristics of patients with NVAF, with or without history of cancer. The mean (±SD) and proportions (percentage) were calculated for continuous and categorical variables, respectively. Patients with AF and cancer and those without cancer were stratified according to their stroke and bleeding risk, and then we estimated the proportion of patients and 95% CI in each stratum.

Survival (time‐to‐event) models were constructed to compare time to the predefined outcome for patients with NVAF and history of cancer versus the referent group (patients with NVAF and no cancer). Time‐to‐event was defined as the time between the index date (NVAF diagnosis) to the earliest date among the event of interest, or censoring date. Patients were censored at their end of follow‐up, and if they developed any of the 5 cancer types after AF diagnosis (for patients with no cancer history). Parametric proportional hazard survival models, including selected variables, were estimated. Exponential, Weibull proportional hazard models were performed and reporting adjusted hazard ratios (aHRs) and 95% CI of the events of interest (stroke and major bleeding). This analysis indicated the relative hazard of developing the endpoint upon exposure to each type of cancer diagnosis (breast, prostate, colorectal, hematological, or lung cancer) versus the referent group (patients with AF and no history of cancer). All models were adjusted for all the covariates mentioned earlier, including chronic conditions, exposure to aspirin or OAC as time‐varying covariates, and did take into account clustering by general practice. Missing BMI values at baseline were imputed by an interpolation algorithm that has been used in previous studies using the CPRD.^24^ A previously used algorithm was also used to manage smoking status inconsistencies at baseline.^25^ The main model that estimated the risk of ischemic stroke, included patients from all CHA_2_DS_2_‐VASc risk categories, and 2 additional models included subgroups of (1) patients with low and intermediate stroke risk, and (2) only patients with high stroke risk. Similarly, the main model that estimated the risk of major bleeding events, included patients from all HAS‐BLED risk categories, and 2 additional models included subgroups of (1) patients with low and intermediate bleeding risk, and (2) only patients with high bleeding risk.

To investigate the effect of the length of time from cancer diagnosis to NVAF diagnosis on the risk of stroke or bleeding, we conducted 2 sensitivity analyses. In the first, cancer was defined as active if it occurred 6 months before NVAF, and in the second, if it occurred 5 years before NVAF. A third sensitivity analysis included stroke events recorded in the HES database alone, ie, excluding stroke records from primary care. Similar to the main model, parametric hazard survival models were used in all sensitivity analyses, adjusted for the variables previously described. All tests were 2‐sided and an alpha level of 0.05 was used throughout. All analyses were performed using Stata 16 (StataCorp LP, College Station, TX).

## RESULTS

### Participants

**Figure 2:**
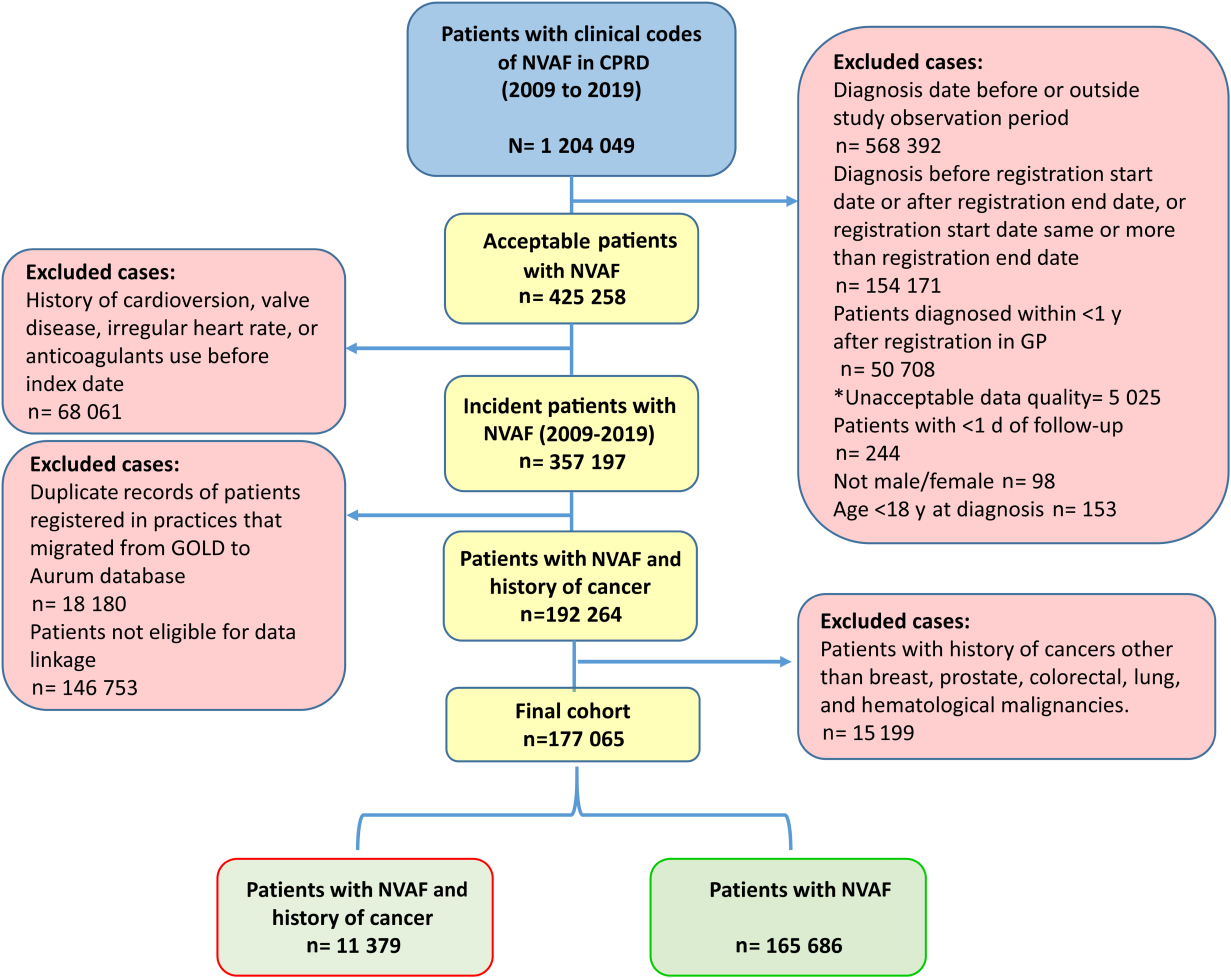
Cohort selection and number of included and excluded patients. NVAF indicates nonvalvular atrial fibrillation; CPRD, clinical practice datalink; and GP, general practice. *Records that are deemed to be not of research quality (inconsistent or incomplete recording of patient data).

**Figure 3:**
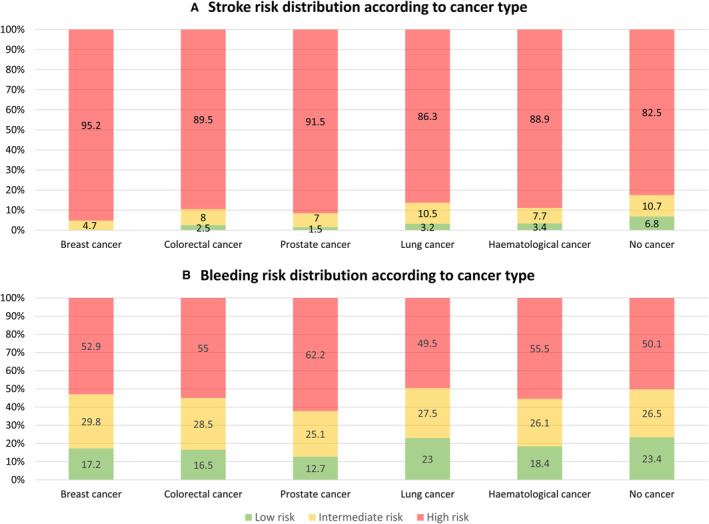
Stroke and bleeding risk distribution at baseline in patients with atrial fibrillation based on cancer type. **A,** Stratified by CHA_2_DS_2_‐VASc score, which consists of 8 categories, with points given for each of the following: congestive heart failure, hypertension, age ≥75 years (×2 points), diabetes mellitus, prior stroke or transient ischemic attack (TIA) or thromboembolism (×2 points), vascular disease, age 65 to 74 years, and sex category. **B**, Modified HAS‐BLED score, which consists of 8 points, one for each of the following: hypertension, abnormal kidney or liver function (1 point each), stroke, history of bleeding or predisposition, labile INR, elderly (>65 years), and drugs/alcohol concomitantly (1 point each).

During the study window of 11 years, 177 065 patients satisfied the inclusion and exclusion criteria (Figure 2). The study cohort comprised of 165 686 (93.6%) patients with NVAF without cancer and 11 379 (6.4%) diagnosed with cancers of interest within 2 years before incident NVAF at baseline. The mean (SD) age of patients with AF and cancer was 78.0 (9.0) and 74.4 (12.6) years, respectively in patients without a history of cancer. In the cancer group, around 64.8 of patients were men, and prostate cancer was the most prevalent cancer at 34.8% followed by hematological cancer (21.9%), breast cancer (14.9%), colorectal cancer (14.6%), and lung cancer (13.8%), respectively (Table). Most patients with cancer (67.8%) had high level of comorbidities (Charlson comorbidity index ≥3), as for patients with no cancer 50 302 (30.4%) had a Charlson comorbidity index ≥3. Overall, polypharmacy and baseline comorbidities were more frequent in patients with NVAF and a history of cancer than in patients with AF only; polypharmacy 54.9% versus 46.1%, hypertension 65.8% versus 63.3%, ischemic heart disease 31.8% versus 28.9%, chronic kidney disease 27.4% versus 22.4%, and anemia 25.2% versus 16% respectively. According to baseline CHA_2_DS_2_‐VASc score, stroke risk distribution varied across cancer types compared with patients with no cancer. High risk for stroke as stratified by CHA_2_DS_2_‐VASc score was more common in patients with cancer at around 90.5% compared with patients with no history of cancer (82.5%) (Figure 3). Similarly, high bleeding risk as stratified by HAS‐BLED score was more common among patients with cancer at around 56.5%, compared with patients with no history of cancer (50.1%).

**Table:** Patient Characteristics According to Cancer Status

Baseline characteristics	Cancer and NVAF	NVAF only	*P* value
	n = 11 379	n = 165 686	
Male sex, n (%)	7382 (64.8)	87 243 (52.6)	<0.001
Age, y, mean (SD)	78.0 (9.0)	74.4 (12.6)	<0.001
BMI, mean (SD)	27.2 (5.4)	28.3 (6.1)	<0.001
Ethnicity, n (%)			
White	10 904 (95.8)	152 138 (91.8)	<0.001
Black	136 (1.2)	1446 (0.9)	
Asian	99 (0.9)	2376 (1.4)	
Other ethnicities	72 (0.6)	1472 (0.9)	
Unknown	168 (1.5)	8254 (5.0)	
IMD quintile, n (%)			
IMD 1 (least deprived)	2830 (24.9)	40 677 (24.6)	<0.001
IMD 2	2520 (22.2)	37 122 (22.4)	
IMD 3	2346 (20.6)	34 193 (20.6)	
IMD 4	1993 (17.5)	29 192 (17.6)	
IMD 5 (most deprived)	1690 (14.8)	24 502 (14.8)	
Charlson comorbidity index, n (%)			
0	353 (3.1)	55 396 (33.4)	<0.001
1 or 2	3315 (29.1)	59 988 (36.2)	
≥3	7711 (67.8)	50 302 (30.4)	
Baseline comorbidities, n (%)			
Diabetes	2607 (22.9)	35 013 (21.1)	<0.001
Hypertension	7483 (65.8)	104 833 (63.3)	<0.001
Cerebrovascular disease/TIA	1797 (15.8)	24 870 (15.0)	<0.001
Heart failure	1377 (12.1)	16 332 (9.9)	<0.001
Ischemic heart disease	3617 (31.8)	47 953 (28.9)	<0.001
Myocardial infarction	1248 (10.9)	16 031 (9.7)	0.006
Peripheral vascular disease	789 (6.9)	8781 (5.3)	<0.001
Chronic obstructive pulmonary disease	2314 (20.3)	24 642 (14.9)	<0.001
Chronic kidney disease	3113 (27.4)	37 076 (22.4)	<0.001
Liver disease	96 (0.8)	1483 (0.9)	0.818
Anemia	2863 (25.2)	26 495 (16.0)	<0.001
Peptic ulcer	829 (7.3)	8617 (5.2)	<0.001
History of falls	1714 (15.1)	21 555 (13.0)	<0.001
Dementia	371 (3.3)	6090 (3.7)	0.003
Baseline treatment, n (%)			
Aspirin	3730 (32.8)	56 101 (33.8)	0.521
Antiplatelet	1098 (9.6)	15 638 (9.4)	0.235
NSAIDs	804 (7.1)	11 900 (7.2)	0.220
Statins	4793 (42.1)	69 418 (41.9)	0.052
PPI	4265 (37.5)	48 508 (29.3)	<0.001
CCB	190 (1.7)	3110 (19.)	0.199
Corticosteroids	1452 (12.8)	13 075 (7.9)	<0.001
SSRI/SNRI	844 (7.4)	12 208 (7.4)	0.189
Polypharmacy	6255 (54.9)	76 435 (46.1)	<0.001
Stroke risk			
CHA_2_DS_2_‐VASc* score, mean (SD)	3.4 (1.6)	3.2 (1.8)	<0.001
Low risk, n (%)	237 (2.1)	11 216 (6.8)	<0.001
Intermediate risk, n (%)	847 (7.4)	17 837 (10.8)	
High risk, n (%)	10 295 (90.5)	136 633 (82.5)	
Bleeding risk			
HAS‐BLED$ score (mean)	2.7 (1.3)	2.5 (1.3)	<0.001
Low risk, n (%)	1890 (16.6)	38 789 (23.4)	<0.001
Intermediate risk, n (%)	3056 (26.9)	43 842 (26.5)	
High risk, n (%)	6433 (56.5)	83 055 (50.1)	
Cancer type, n (%)			
Breast cancer	1691 (14.9)	NA	
Prostate cancer	3955 (34.8)		
Colorectal cancer	1666 (14.6)		
Hematological cancer	2491 (21.9)		
Lung cancer	1576 (13.8)		
Chemotherapy (in prior 3 mo), n (%)	697 (6.1)		

NVAF indicates nonvalvular atrial fibrillation; BMI, body mass index; CCB, calcium channel blocker; IMD, index of multiple deprivation; IQR, interquartile range; NSAID, nonsteroidal anti‐inflammatory drug; PPI, proton pump inhibitor; SNRI, selective norepinephrine reuptake inhibitor; SSRI, selective serotonin reuptake inhibitor; and TIA, transient ischemic attack.

* Consists of 8 points, with points given for each of the following: congestive heart failure, hypertension, age ≥75 years (×2 points), diabetes mellitus, prior stroke or TIA or thromboembolism (×2 points), vascular disease, age 65 to 74 years, and sex category (female). $Modified HAS‐BLED consists of 8 points, one point for each of the following: hypertension, abnormal kidney or liver function (1 point each), stroke, history of bleeding or predisposition, elderly (>65 years), and drug (antiplatelets or NSAIDs) or alcohol consumption.

### Outcome Events

**Figure 4:**
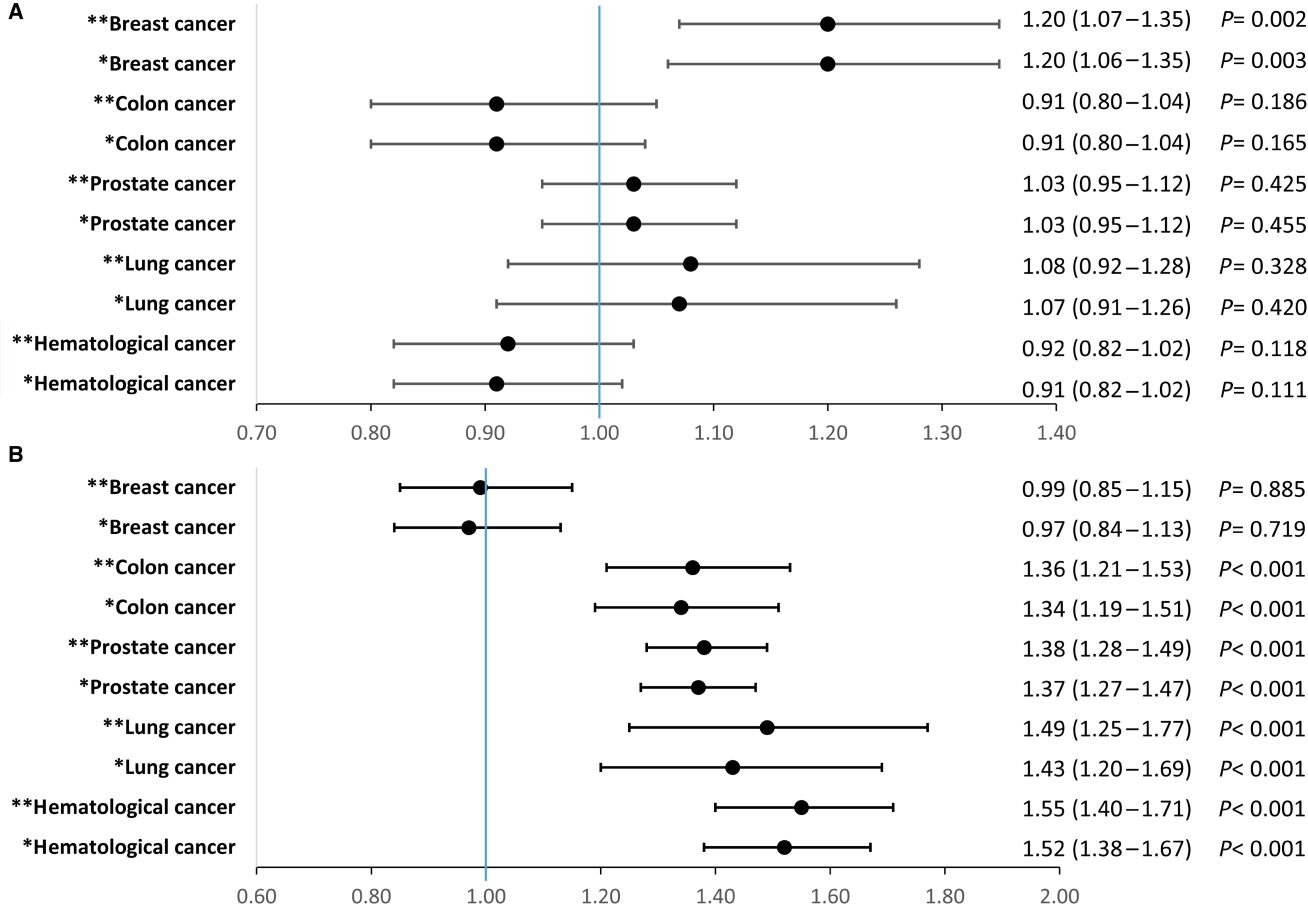
Forest plots describing adjusted hazard ratios (aHRs) and 95% CI associated with cancer and the event of interest. **A**, aHRs associated with cancer type for developing ischemic stroke. **B**, aHRs associated with cancer type for developing major bleeding events. *Estimates were adjusted for sex, age, smoking, alcohol consumption, body mass index. Index of multiple deprivation and other time varying covariates such as diabetes, hypertension, ischemic heart disease, dementia, and heart failure. **Estimates were adjusted for sex, age, smoking, alcohol consumption, body mass index, index of multiple deprivation and other time varying covariates such as diabetes, hypertension, ischemic heart disease, dementia, heart failure, and exposure to oral anticoagulants and aspirin.

In patients with no history of cancer, median (25th–75th centile) follow‐up time was 2.3 years (0.8–4.6), while it was 1.3 years (0.4–3.1) for patients with cancer. The crude incidence rate of ischemic stroke per 100 person‐years at risk (95% CI) for patients with cancer were: 8.4 (95% CI, 7.5–9.3) for breast cancer, 6.7 (95% CI, 6.0–7.6) for colorectal cancer, 8.2 (95% CI, 7.7–8.9) for prostate cancer, 9.5 (95% CI, 8.2–11.1) for lung cancer, and 7.4 (95% CI, 6.6–8.1) for hematological cancer, compared with 6.1 per 100 person‐years at risk (95% CI, 6.0–6.2) among patients with no cancer. Crude hazard ratios for stroke risk are described in Table S1. After adjusting for the differences in baseline covariates, breast cancer was found to have higher risk of stroke compared with no cancer (aHR, 1.20 [95% CI, 1.07–1.35]) (Figure 4A; Table S2). This was also consistent with the sensitivity analysis that considered stroke events from HES records only (Table S3), the model that considered patients with high risk for stroke (Table S4), and the model that included cancer diagnosed within 5 years before AF (Table S5). The sensitivity analysis that included patients with low/intermediate stroke risk found no significant association between cancer and stroke (Table S4). However, the sensitivity analysis that considered the length of time of cancer diagnosis before NVAF, found that prostate cancer was associated with higher stroke risk compared with no cancer if diagnosed within 6 months before NVAF (aHR, 1.11 [95% CI, 1.01–1.22]) (Table S4). In the model that included all patients with cancer diagnosed at any time point before AF found no increase in risk of stroke associated with cancer (Table S5). Finally, the model that combined all patients with cancer into 1 group also found that there is no increase in stroke risk when compared with patients without cancer (Table S6).

The crude incidence rate of major bleeding events among patients with cancer were: 5.0 per 100 person‐years at risk (95% CI, 4.3–5.7) for breast cancer, 8.7 (95% CI, 7.8–9.7) for colorectal cancer, 10.2 (95% CI, 9.6–10.9) for prostate cancer, 9.5 (95% CI, 8.1–11.1) for lung cancer, and 9.5 (95% CI, 8.7–10.4) for hematological cancer, compared with 5.1 per 100 person‐years at risk (95% CI, 5.0–5.1) for patients without cancer. Crude hazard ratios for bleeding risk are described in Table S7. After adjusting for the differences in baseline covariates and exposure to OAC or aspirin (Figure 4B), high bleeding risk was observed in hematological cancer (aHR, 1.55 [95% CI, 1.40–1.71]), lung cancer (aHR, 1.49 [95% CI, 1.25–1.77]), prostate cancer (aHR, 1.38 [95% CI, 1.28–1.49]), and colorectal cancer (aHR, 1.36 [95% CI, 1.21–1.53]). In contrast, there was no increased risk of major bleeding events for patients with breast cancer, compared with patients with no cancer (aHR, 0.99 [95% CI, 0.85–1.15]). (Figure 4B; Table S8). In the sensitivity analysis that considered bleeding risk category (Table S9), risk differences between cancer and patients without cancer varied across low/intermediate‐ and high‐risk bleeding groups. Patients with hematological malignancies, prostate, and colorectal cancers had higher risk of bleeding, relative to patients without cancer, if they were at low or intermediate risk of bleeding.

In the sensitivity analysis models (Table S10), cancer was defined as active if it occurred either 6 months before AF, 2 years before NVAF, or 5 years before AF. Across all the 3 models, results were consistent with the fully adjusted model in Figure 4B, although higher hazard ratios observed in patients diagnosed with cancer 6 months before AF diagnosis. Overall, in the model that combined all cancer types into 1 category found cancer in general to have increased risk of bleeding compared with patients without cancer (aHR, 1.37 [95% CI, 1.30–1.44]) (Table S11).

## DISCUSSION

In this population‐based cohort study of patients with NVAF, we identified that a recent history of breast cancer or prostate cancer was associated with an increased risk of ischemic stroke, after adjusting for differences in baseline characteristics. Moreover, a history of hematological, lung, prostate, or colorectal cancers was associated with increased risk of major bleeding events, even in the absence of treatment with oral anticoagulant or antiplatelet agents. Finally, the closer the temporal relationship between cancer and AF diagnosis, the higher the risk of major bleeding events.

Cancer and AF both can commonly occur later in life. The association between cancer and AF is not surprising, since both conditions share risk factors, such as male sex, older age, obesity, COPD, liver disease, hypertension, and diabetes. We have confirmed in this study that patients with NVAF with history of cancer are generally older, and more likely to suffer from more chronic conditions than those with no history of cancer. Prior studies have shown that the incidence rate of AF in the cancer population is higher than in the general population,^16^ and previous CPRD studies that reported cancer prevalence in the AF population showed an overall prevalence of 15% to 21%,^26^, ^27^, ^28^, ^29^ which is similar to what we observed, considering the number of excluded patients with history of other cancer types. Our results have shown that almost 1 in every 10 patients with NVAF has a history of cancer, although this is likely to underestimate the true burden of cancer in this population as we have only focused on 5 types of cancer. Earlier reports on the prevalence of cancer in AF population showed an overall prevalence of around 20%,^29^, ^30^ which is similar to what we observed, considering the number of excluded patients with history of other cancer types. Moreover, our analysis was based on a general practice database that is broadly representative of the UK population, and validation of CPRD data has shown high‐positive predictive value of some diagnoses and, comparisons of incidence with other data sources from the UK are also broadly similar.^13^, ^14^

Prior observational studies that examined the association between cancer diagnosis and subsequent stroke risk, reported that cancer increases the risk of stroke, although this association depends on the type of cancer and time of cancer diagnosis before stroke occurrence.^31^, ^32^ Stroke events associated with cancer arise from unique mechanisms linked to cancer‐mediated hypercoagulability, or complications of oncological therapies.^31^, ^32^ These studies investigated the risk of stroke in the general cancer population without focusing on specific cardiovascular conditions. However, results from a Swedish cohort study, that included patients with AF and history of cancer,^33^ showed no increase in cardioembolic stroke risk in patients with cancer. In contrast, our analysis showed that the crude incidence of ischemic stroke among patients with cancer in general was higher than in patients without a history of cancer, with the highest rates observed in patients with lung cancer. After accounting for additional risk factors and confounders (patient characteristics, comorbidities, and exposure to OAC/aspirin), this association was significant for breast cancer and for prostate cancer, for the latter only when recently diagnosed (6 months before NVAF). Although, previous studies of cancers associated with increased stroke risk reported contradictory findings,^31^, ^32^, ^33^ some studies showed high incidence of stroke in patients with breast and prostate cancer.^34^, ^35^

We report that the incidence rate of major bleeding events was overall higher among patients with a history of cancer, compared with patients without cancer, except for breast cancer. These associations were statistically significant even after accounting for possible risk factors for bleeding (eg, exposure to OAC/aspirin). In our analysis, the highest bleeding risk was associated with hematological cancer, followed by lung cancer, prostate, and colorectal cancer. Information on the cancer type associated with the highest risk of bleeding can be inconsistent across studies, because of the different type of cancers included and how each study defines a certain cancer type. However, similar to our findings, previous observational studies on the effect of anticoagulation on bleeding risk among patients with AF with cancer,^36^, ^37^ have reported an increased risk of major bleeding, associated with lung cancer. The fact that lung cancer was associated with high bleeding risk is not surprising, since lungs contain an extensive network of pulmonary capillaries, and one of the common complications of lung cancer is bleeding in the airway causing haemoptysis.^38^ Whereas the high bleeding risk observed with both hematological and other higher stage malignancies can be explained by alterations in platelet function and numbers, deficiencies in clotting factors, circulating anticoagulants, and defects in vascular integrity, that collectively increase bleeding risk.^18^ Although the increase in bleeding risk observed in our analysis can be partially explained in theory by these biological mechanisms, it's still not fully understood how specific cancer types alter stroke and bleeding risks in patients with AF.

When considering this is the context of a similar risk of stroke between patients with AF with and without cancer, it raises the question of the net clinical benefit of anticoagulation for patients with AF and cancer. Because of the increase in bleeding risk found in our analysis and in previous studies for patients with cancer,^36^, ^37^ the net clinical benefit of anticoagulation is likely to be different compared with patients without cancer. If the incidence of cardioembolic stroke is confirmed to be not high, OAC may actually be harmful in certain patients with certain types of cancer (especially those without high risk of ischemic stroke). On the other hand, patients with AF at risk for stroke may place greater value on preventing stroke than avoiding bleeding events, because of the long‐term impact of stroke compared with bleeding. Therefore, if the balance between stroke and bleeding risk is altered by certain types of cancer, it may not translate into changing the decisions about anticoagulation therapy, especially when considering patients stroke risk, and patient preference. However, an earlier study reported that there are no truly low‐risk patients with cancer,^39^ and there is insufficient evidence on the net OAC benefits for patients with cancer and AF, with a CHA_2_‐DS_2_‐VASc score between 0 and 1. Nevertheless, if cancer is assumed to moderately increase stroke or bleeding risks, including it as a factor in risk predicting scores might not have a noticeable impact in altering the balance between stroke and bleeding risk. This is because previous studies that examined the predictive accuracy of CHA_2_‐DS_2_‐VASc score have found that the score performs modestly in predicting ischemic stroke overall,^40^ and its role is primarily to help distinguish patients with AF who require anticoagulation.

The study also has several strengths. To our knowledge, this is the first study to describe the association between NVAF in patients with cancer with the risk of ischemic stroke and bleeding events in England using electronic health records collected routinely in primary care. First, we used 2 different data sources, the CPRD GOLD and Aurum databases, which enabled inclusion of a large sample that is representative of the population of England,^13^, ^14^ and were both linked to HES data and the ONS death registry data to maximize capture of recorded events and ascertain occurrence of outcomes. Second, we applied conservative inclusion criteria to only include newly diagnosed patients with NVAF, which in case of patients with cancer the type of AF they devolved could be secondary to cancer and this increased the validity of the presented results. Third, we used advanced multiple imputation technique to impute missing body mass index values, and we also adjusted for important clinical time‐varying risk factors such as chronic conditions and exposure to OAC/aspirin. Fourth, the supplementary analyses confirmed the robustness of the findings on different stroke/bleeding risk categories, and duration of cancer history before NVAF.

### Study Limitations

This study has limitations. First, as this study was not conducted using cancer registration data, but mainly by using a primary care database (albeit supplemented with national hospital admissions data to minimize misclassification), data specific to cancer management may not be complete (eg, cancer stage, and systemic anti‐cancer therapy/radiotherapy records) therefore, residual confounding is likely to remain. Second, prescribed medication data were analyzed using primary records only and if patients received care exclusively in hospital settings, then we would incorrectly define them as untreated because of lack of prescription data in secondary care records. There is also the possibility of over‐the‐counter usage of aspirin which we could capture and assess in our analysis; however, according to a previous CPRD study that compared prescription records of aspirin to patient self‐report have found that the majority of chronic aspirin therapy were captured by CPRD records and the amount of over‐the‐counter use of aspirin were likely to be low.^41^ Third, our findings are dependent on accurate recording from the health professionals. Lack of event recording would result in a false‐negative classification of a certain event and therefore could potentially bias our findings. However, considering the clinical significance of the event (ie, AF or cancer diagnosis) we would expect recording for these events to be relatively complete, especially since quality indicators for this population are incentivized in English primary care through the Quality and Outcomes Framework.^42^, ^43^ Fourth, although we have identified an association between certain cancer types and stroke or bleeding risk, we were not able to establish the true mechanism the drives certain cancer types to influence bleeding and stroke risk. Mainly because of the nature of this study being, an observational retrospective cohort based on primary care records not linked to a cancer registry. Therefore, we recommend careful interpretation of the study results.

## CONCLUSIONS

This population‐based cohort of patients with cancer and NVAF observed that in patients with certain types of cancer (breast and prostate cancer), the risk of ischemic stroke was increased when compared with patients with no cancer history. In contrast, the risk of major bleeding was increased in patients with hematological, lung, colorectal, and prostate cancer, particularly in those with a recent diagnosis (<6 months) of cancer. Our analysis highlights the challenges in the prescription of oral anticoagulant therapy in this cohort of patients, particularly when net clinical benefit is unclear.

## Sources of Funding

A.M.A. research was supported by the Ministry of Education of Saudi Arabia as part of a PhD scholarship at The University of Manchester. D.M.A. is funded by the National Institute of Health and Care Research Greater Manchester Patient Safety Research Collaboration (PSRC‐2016‐003). The funders had no role in study design, data collection and analysis, decision to publish, or preparation of the manuscript.

## Disclosures

D.M.A. received research grants from AbbVie, Almirall, Celgene, Eli Lilly, Janssen, Novartis, UCB, and the Leo Foundation. The remaining authors have no disclosures to report.

## Supporting information

Tables S1–S11
