# Correction to: Common Cancer Types and Risk of Stroke and Bleeding in Patients With Nonvalvular Atrial Fibrillation: A Population‐Based Study in England

**DOI:** 10.1161/JAHA.123.027748

**Published:** 2023-12-18

In the article by Alyaa M. Ajabnoor et al, “Common Cancer Types and Risk of Stroke and Bleeding in Patients With Nonvalvular Atrial Fibrillation: A Population‐Based Study in England,” which published on September 26, 2023 (*J Am Heart Assoc*. 2023;12:e029423. DOI: 10.1161/JAHA.123.029423) and was included in the October 3, 2023 issue of the journal, a correction was needed.

The second paragraph of the Discussion section was duplicated. This has been corrected.

The authors regret the error.

The online version of the article has been updated and is available here: https://www.ahajournals.org/doi/full/10.1161/JAHA.123.029423.

