# Supplementary material for: Common Cancer Types and Risk of Stroke and Bleeding in Patients With Nonvalvular Atrial Fibrillation: A Population‐Based Study in England
Source: J Am Heart Assoc. 2023 Sep 26;12(19):e029423. doi: 10.1161/JAHA.123.029423 (PMC10727261; doi:10.1161/JAHA.123.029423)
Supplement: Supplementary file 1 — Tables S1–S11 [file JAH3-12-e029423-s001.pdf]

SUPPLEMENTAL MATERIAL

Table S1. Crude hazard ratios and 95% confidence interval associated with cancer type for developing ischaemic stroke.

	Crude HRs 95%CI	P value
**Cancer type**		
No cancer	Ref	
Breast cancer	1.17 (1.02 - 1.34)	0.023
Colorectal cancer	1.03 (0.88 - 1.21)	0.717
Prostate cancer	1.06 (0.94 - 1.20)	0.351
Lung cancer	0.58 (0.46 - 0.74)	0.000
Haematological cancer	0.81 (0.69 - 0.95)	0.010

Table S2. Adjusted hazard ratios for time varying covariates and 95% confidence interval associated with cancer type for developing ischaemic stroke.

	Adjusted HR 95%CI	P value
**Cancer type**		
No cancer	Ref	
Breast cancer	1.20 (1.07; 1.35)	0.002
Colorectal cancer	0.91 (0.80; 1.04)	0.186
Prostate cancer	1.03 (0.95; 1.12)	0.425
Lung cancer	1.08 (0.92; 1.28)	0.328
Haematological cancer	0.92 (0.82; 1.02)	0.118
**Patient level IMD**		
1 (least deprived)	Ref	
2	1.05 (1.01; 1.09)	0.006
3	1.06 (1.02; 1.09)	0.003
4	1.10 (1.06; 1.14)	<0.001
5 (most deprived)	1.19 (1.14; 1.23)	<0.001
**Sex**		
Male	Ref	
Female	1.05 (1.03; 1.08)	<0.001
**Baseline age and BMI**		
Age	1.036 (1.034; 1.037)	<0.001
BMI	0.990 (0.987; 0.992)	<0.001
**Disease state and treatment**		
Ischaemic heart disease	1.03 (1.00; 1.06)	0.036
Heart failure	1.04 (1.01; 1.08)	0.010
Dementia	1.61 (1.53; 1.68)	<0.001
Diabetes	1.15 (1.12; 1.18)	<0.001
Hypertension	1.08 (1.05; 1.12)	<0.001
Myocardial infarction	1.06 (1.02; 1.10)	0.002
OAC (warfarin or NOAC)	1.13 (1.10; 1.17)	<0.001
Aspirin	0.96 (0.93; 1.00)	0.032
History of stroke	3.49 (3.40; 3.59)	<0.001
Metastasis	1.03 (0.93; 1.15)	0.569
**Smoking**		
Non-smoker	Ref	
Current/ex-smoker	1.14 (1.10; 1.17)	<0.001
**Alcohol consumption**		
No alcohol consumption	Ref	
Light drinker	0.94 (0.90; 0.98)	0.007
Former drinker	0.97 (0.88; 1.07)	0.495
Moderate drinker	0.92 (0.89; 0.96)	<0.001
Heavy drinker	1.01 (0.97; 1.06)	0.576

Table S3. Adjusted hazard ratios for time varying covariates and 95% confidence interval associated with cancer type for developing ischaemic stroke. (Stroke events from HES records only)

	Adjusted HR 95%CI	P value
**Cancer type**		
No cancer	Ref	
Breast cancer	1.16 (1.01; 1.33)	0.040
Colorectal cancer	0.86 (0.73; 1.01)	0.062
Prostate cancer	0.96 (0.88; 1.06)	0.440
Lung cancer	1.13 (0.94; 1.37)	0.189
Haematological cancer	0.96 (0.85; 1.09)	0.564
**Patient level IMD**		
1 (least deprived)	Ref	
2	1.04 (1.00; 1.08)	0.075
3	1.08 (1.04; 1.13)	<0.001
4	1.15 (1.10; 1.20)	<0.001
5 (most deprived)	1.20 (1.15; 1.26)	<0.001
**Sex**		
Male	Ref	
Female	1.05 (1.02; 1.08)	0.001
**Baseline age and BMI**		
Age	1.049 (1.047; 1.050)	<0.001
BMI	0.991 (0.988; 0.993)	<0.001
**Disease state and treatment**		
Ischaemic heart disease	1.04 (1.01; 1.08)	0.010
Heart failure	1.09 (1.05; 1.13)	<0.001
Dementia	1.68 (1.60; 1.77)	<0.001
Diabetes	1.19 (1.15; 1.23)	<0.001
Hypertension	1.15 (1.11; 1.19)	<0.001
Myocardial infarction	1.14 (1.09; 1.19)	<0.001
OAC (warfarin or NOAC)	1.07 (1.03; 1.11)	<0.001
Aspirin	1.03 (0.99; 1.07)	0.151
History of stroke	2.45 (2.38; 2.53)	<0.001
Metastasis	1.00 (0.88; 1.14)	0.956
**Smoking**		
Non-smoker	Ref	
Current/ex-smoker	1.17 (1.13; 1.22)	<0.001
**Alcohol consumption**		
No alcohol consumption	Ref	
Light drinker	0.91 (0.87; 0.96)	<0.001
Former drinker	1.03 (0.93; 1.15)	0.551
Moderate drinker	0.87 (0.83; 0.90)	<0.001
Heavy drinker	1.01 (0.96; 1.07)	0.694

Table S4. Adjusted hazard ratios for time varying covariates and 95% confidence interval associated with cancer type for developing ischaemic stroke based on stroke risk category.

	Stroke in all CHA_2DS_2VASc categories		Stroke in low and intermediate stroke risk patients		Stroke in high stroke risk patients	
**Cancer type**	**Adjusted* HR 95%CI**	**P value**	**Adjusted* HR 95%CI**	**P value**	**Adjusted* HR 95%CI**	**P value**
**No cancer**	Ref					
**Breast cancer**	1.20 (1.07; 1.35)	0.002	0.89 (0.51; 1.58)	0.708	1.22 (1.08: 1.37)	0.002
**Colorectal cancer**	0.91 (0.80; 1.04)	0.186	1.01 (0.58; 1.74)	0.984	0.90 (0.79; 1.04)	0.152
**Prostate cancer**	1.03 (0.95; 1.12)	0.425	0.79 (0.51; 1.23)	0.302	1.05 (0.96; 1.13)	0.283
**Lung cancer**	1.08 (0.92; 1.28)	0.328	1.17 (0.68; 2.01)	0.574	1.05 (0.89; 1.26)	0.548
**Haematological cancer**	0.92 (0.82; 1.02)	0.118	0.95 (0.60; 1.49)	0.810	0.91 (0.81; 1.02)	0.105

*Estimates were adjusted for sex, age, smoking, alcohol consumption, BMI. IMD, and other time varying covariates such as; exposure to OAC/aspirin, diabetes, hypertension, IHD, dementia and heart failure

Table S5. Sensitivity analysis for adjusted hazard ratios and 95% confidence interval associated with cancer type for developing ischaemic stroke, based on different time intervals of cancer diagnosis.

	Active cancer - within 6 months before AF		Active cancer- within 2 years before AF		Active cancer- within 5 years before AF		Cancer diagnosed at any time point before AF	
**Cancer type**	**Adjusted HR 95%CI**	**P value**	**Adjusted HR 95%CI**	**P value**	**Adjusted HR 95%CI**	**P value**	**Adjusted HR 95%CI**	**P value**
No cancer	Ref							
Breast cancer	1.15 (0.98; 1.35)	0.096	1.20 (1.07; 1.35)	0.002	1.15 (1.05; 1.27)	0.004	1.04 (0.97; 1.12)	0.242
Colorectal cancer	0.96 (0.81; 1.14)	0.625	0.91 (0.80; 1.04)	0.186	0.91 (0.82; 1.02)	0.101	0.94 (0.86; 1.02)	0.110
Prostate cancer	1.11 (1.01; 1.22)	0.031	1.03 (0.95; 1.12)	0.425	1.02 (0.95; 1.10)	0.558	1.01 (0.94; 1.07)	0.850
Lung cancer	1.15 (0.96; 1.37)	0.137	1.08 (0.92; 1.28)	0.328	1.01 (0.86; 1.19)	0.876	0.99 (0.85; 1.15)	0.885
Haematological cancer	0.98 (0.87; 1.12)	0.816	0.92 (0.82; 1.02)	0.118	0.93 (0.84; 1.03)	0.151	0.88 (0.80; 0.96)	0.005

*Estimates were adjusted for sex, age, smoking, alcohol consumption, BMI. IMD, and other time varying covariates such as; exposure to OAC/aspirin, diabetes, hypertension, IHD, dementia and heart failure

Table S6. Adjusted hazard ratios for time varying covariates and 95% confidence interval associated with all cancer types for developing ischaemic stroke.

	Adjusted HR 95%CI	P value
**Cancer type**		
No cancer	Ref	
All cancer types*	1.02 (0.97; 1.07)	0.510
**Patient level IMD**		
1 (least deprived)	Ref	
2	1.05 (1.01; 1.09)	0.007
3	1.06 (1.02; 1.09)	0.003
4	1.10 (1.06; 1.14)	<0.001
5 (most deprived)	1.19 (1.14; 1.23)	<0.001
**Sex**		
Male	Ref	
Female	1.06 (1.03; 1.08)	<0.001
**Baseline age and BMI**		
Age	1.04 (1.03; 1.04)	<0.001
BMI	0.99 (0.99; 0.99)	<0.001
**Disease state and treatment**		
Ischaemic heart disease	1.03 (1.00; 1.06)	0.041
Heart failure	1.04 (1.01; 1.08)	0.011
Dementia	1.61 (1.54; 1.68)	<0.001
Diabetes	1.15 (1.12; 1.18)	<0.001
Hypertension	1.08 (1.05; 1.12)	<0.001
Myocardial infarction	1.06 (1.02; 1.10)	0.002
OAC (warfarin or NOAC)	1.13 (1.10; 1.16)	<0.001
History of bleeding	0.96 (0.92; 1.00)	0.031
Aspirin	3.49 (3.41; 3.58)	<0.001
Metastasis	1.05 (0.95; 1.17)	0.339
**Smoking**		
Non-smoker	Ref	
Current/ex-smoker	1.14 (1.10; 1.17)	<0.001
**Alcohol consumption**		
No alcohol consumption	Ref	
Light drinker	0.94 (0.90; 0.98)	0.008
Former drinker	0.97 (0.88; 1.06)	0.484
Moderate drinker	0.92 (0.89; 0.96)	<0.001
Heavy drinker	1.01 (0.97; 1.06)	0.584

*includes breast, prostate, colorectal, haematological, and lung cancer.

Table S7. Crude hazard ratios and 95% confidence interval associated with cancer type for developing major bleeding events.

	Crude HRs 95%CI	P value
**Cancer type**		
No cancer	Ref	
Breast cancer	0.95 (0.83; 1.08)	0.443
Colorectal cancer	1.63 (1.47; 1.82)	<0.001
Prostate cancer	1.89 (1.77; 2.02)	<0.001
Lung cancer	1.55 (1.33; 1.81)	<0.001
Haematological cancer	1.72 (1.57; 1.88)	<0.001

Table S8. Adjusted hazard ratios for time varying covariates and 95% confidence interval associated with cancer type for developing major bleeding events.

	Adjusted HR 95%CI	P value
**Cancer type**		
No cancer	Ref	
Breast cancer	0.99 (0.85; 1.15)	0.885
Colorectal cancer	1.36 (1.21; 1.53)	<0.001
Prostate cancer	1.38 (1.28; 1.49)	<0.001
Lung cancer	1.49 (1.25; 1.77)	<0.001
Haematological cancer	1.55 (1.40; 1.71)	<0.001
**Patient level IMD**		
1 (least deprived)	Ref	
2	1.02 (0.98; 1.02)	0.378
3	1.10 (1.06; 1.10)	<0.001
4	1.14 (1.10; 1.14)	<0.001
5 (most deprived)	1.18 (1.13; 1.18)	<0.001
**Sex**		
Male	Ref	
Female	0.74 (0.72; 0.74)	<0.001
**Baseline age and BMI**		
Age	1.035 (1.034; 1.037)	<0.001
BMI	1.001 (0.999; 1.004)	0.304
**Disease state and treatment**		
Ischaemic heart disease	1.14 (1.11; 1.14)	<0.001
Heart failure	1.15 (1.11; 1.15)	<0.001
Dementia	1.40 (1.33; 1.40)	<0.001
Diabetes	1.06 (1.03; 1.06)	<0.001
Hypertension	1.11 (1.08; 1.11)	<0.001
Myocardial infarction	1.11 (1.07; 1.11)	<0.001
OAC (warfarin or NOAC)	1.82 (1.76; 1.82)	<0.001
History of bleeding	1.23 (1.18; 1.23)	<0.001
Aspirin	1.30 (1.26; 1.30)	<0.001
Metastasis	1.22 (1.09; 1.22)	<0.001
**Smoking**		
Non-smoker	Ref	
Current/ex-smoker	1.12 (1.08; 1.12)	<0.001
**Alcohol consumption**		
No alcohol consumption	Ref	
Light drinker	0.93 (0.88; 0.93)	0.002
Former drinker	1.12 (1.01; 1.12)	0.034
Moderate drinker	0.87 (0.84; 0.87)	<0.001
Heavy drinker	0.99 (0.94; 0.99)	0.612

Table S9. Adjusted hazard ratios for time varying covariates and 95% confidence interval associated with cancer type for developing major bleeding events based on bleeding risk category.

	Bleeding events in all HASBLED categories		Bleeding in low and intermediate bleeding risk patients		Bleeding in high bleeding risk patients	
**Cancer type**	**Adjusted HR* 95%CI**	**P value**	**Adjusted HR* 95%CI**	**P value**	**Adjusted HR* 95%CI**	**P value**
**No cancer**	Ref					
**Breast cancer**	0.99 (0.85; 1.15)	0.885	0.82 (0.63; 1.06)	0.132	1.10 (0.91; 1.32)	0.330
**Colorectal cancer**	1.36 (1.21; 1.53)	<0.001	1.36 (1.11; 1.67)	0.003	1.35 (1.17; 1.56)	<0.001
**Prostate cancer**	1.38 (1.28; 1.49)	<0.001	1.53 (1.35; 1.74)	<0.001	1.31 (1.20; 1.43)	<0.001
**Lung cancer**	1.49 (1.25; 1.77)	<0.001	1.46 (1.11; 1.93)	0.008	1.48 (1.19; 1.84)	<0.001
**Haematological cancer**	1.55 (1.40; 1.71)	<0.001	1.79 (1.53; 2.10)	<0.001	1.41 (1.25; 1.60)	<0.001

*Estimates were adjusted for sex, age, smoking, alcohol consumption, BMI. IMD, and other time varying covariates such as; exposure to OAC/aspirin, diabetes, hypertension, IHD, dementia and heart failure

Table S10. Sensitivity analysis for adjusted hazard ratios and 95% confidence interval associated with cancer type for developing major bleeding events, based on different time intervals of cancer diagnosis.

	Active cancer - within 6 months before AF		Active cancer- within 2 years before AF		Active cancer- with 5 years before AF		Cancer diagnosed at any time point before AF	
**Cancer type**	**Adjusted HR 95%CI**	**P value**	**Adjusted HR 95%CI**	**P value**	**Adjusted HR 95%CI**	**P value**	**Adjusted HR 95%CI**	**P value**
No cancer	Ref							
Breast cancer	1.08 (0.89; 1.31)	0.456	0.99 (0.85; 1.15)	0.885	0.94 (0.84; 1.06)	0.326	0.93 (0.85; 1.01)	0.092
Colorectal cancer	1.46 (1.26; 1.69)	<0.001	1.36 (1.21; 1.53)	<0.001	1.25 (1.13; 1.38)	<0.001	1.18 (1.09; 1.28)	<0.001
Prostate cancer	1.40 (1.28; 1.53)	<0.001	1.38 (1.28; 1.49)	<0.001	1.37 (1.28; 1.46)	<0.001	1.34 (1.27; 1.43)	<0.001
Lung cancer	1.57 (1.31; 1.88)	<0.001	1.49 (1.25; 1.77)	<0.001	1.48 (1.27; 1.73)	<0.001	1.50 (1.30; 1.73)	<0.001
Haematological cancer	1.64 (1.47; 1.84)	<0.001	1.55 (1.40; 1.71)	<0.001	1.50 (1.37; 1.64)	<0.001	1.40 (1.29; 1.53)	<0.001

*Estimates were adjusted for sex, age, smoking, alcohol consumption, BMI. IMD, and other time varying covariates such as; exposure to OAC/aspirin, diabetes, hypertension, IHD, dementia and heart failure

Table S11. Adjusted hazard ratios for time varying covariates and 95% confidence interval associated with all cancer types for developing major bleeding events.

	Adjusted HR 95%CI	P value
**Cancer type**		
No cancer	Ref	
All cancer types*	1.37 (1.30; 1.44)	<0.001
**Patient level IMD**		
1 (least deprived)	Ref	
2	1.02 (0.98; 1.06)	0.369
3	1.10 (1.06; 1.14)	<0.001
4	1.14 (1.10; 1.19)	<0.001
5 (most deprived)	1.18 (1.13; 1.23)	<0.001
**Sex**		
Male	Ref	
Female	0.73 (0.71; 0.75)	<0.001
**Baseline age and BMI**		
Age	1.035 (1.034; 1.037)	<0.001
BMI	1.001 (0.999; 1.004)	0.317
**Disease state and treatment**		
Ischaemic heart disease	1.14 (1.11; 1.18)	<0.001
Heart failure	1.15 (1.11; 1.19)	<0.001
Dementia	1.40 (1.33; 1.48)	<0.001
Diabetes	1.06 (1.03; 1.09)	<0.001
Hypertension	1.11 (1.08; 1.15)	<0.001
Myocardial infarction	1.11 (1.07; 1.15)	<0.001
OAC (warfarin or NOAC)	1.82 (1.76; 1.87)	<0.001
History of bleeding	1.23 (1.18; 1.28)	<0.001
Aspirin	1.30 (1.26; 1.35)	<0.001
Metastasis	1.18 (1.06; 1.32)	0.003
**Smoking**		
Non-smoker	Ref	
Current/ex-smoker	1.12 (1.08; 1.15)	<0.001
**Alcohol consumption**		
No alcohol consumption	Ref	
Light drinker	0.93 (0.88; 0.97)	0.002
Former drinker	1.12 (1.01; 1.24)	0.030
Moderate drinker	0.87 (0.84; 0.91)	<0.001
Heavy drinker	0.99 (0.94; 1.04)	0.614

*includes breast, prostate, colorectal, haematological, and lung cancer.
